# Late migration of silicon as a complication to breast transplant rupture: Case report and literature review

**DOI:** 10.1016/j.ijscr.2021.106241

**Published:** 2021-07-27

**Authors:** Elham Khakbaz, Christian Lang, Giedrius Lelkaitis, Christian Grønhøj

**Affiliations:** a Department of Head and Neck Surgery, Rigshospitalet, Denmark; b Department of Plastic Surgery, Herlev University Hospital, Denmark; c Department of Pathology, Rigshospitalet, Denmark; d Department of Otorhinolaryngology and Maxillofacial Surgery, Zealand University Hospital, Køge, Denmark

**Keywords:** Case report, Silicone implants, Rupture, Migration, Lymphadenopathy

## Abstract

Silicone implants have been used for breast augmentations, both cosmetically and in reconstructive surgery since the 1960s. Rupture of breast implants and silicone migration is a well-known complication. In this case report and literature review, we present a case of a 53-year-old woman with bilateral cosmetic silicone gel breast implant in 1986, and a replacement with saline gel in 2005. The patient had no breast complaints and observed no change in breast volume during this period. In 2020, silicone was randomly identified in a right-sided cervical lymph node in an attempt to remove suspicious lymphadenopathy. The source of the silicone is still doubted; that is, it is not known if the silicone originated from the saline implant or the silicone gel implant.

In our literature review, we find that distant migration of silicone and lymphadenopathy have occurred for silicone breast implants although very rare for saline gel breast implants.

## Highlights

• A case of cervical lymphadenopathy caused by silicone breast migration is described.
• The patient had primarily long-standing silicone gel breast implant and has saline breast implant afterwards.
• The origin of the silicone conglomerate in the cervical lymph node remains unknown.

## 1 Background/introduction

Cronin and Gerow developed the first silicone prosthesis in 1961 and performed the first breast augmentation in 1962. The initial silicone breast implants were silicone rubber shells filled with silicone gel of various consistency. Since then, silicone implants have been developed in five generations, improving the quality of the breast implants to minimize the complications such as gel bleeding, rupture and cosmetic appearance [1]. In general, the prevalence of rupture increases with implant age, and the median life expectancy of silicone implants is 10–16 years [2], [3].

According to a population-based study, the prevalence of silicone breast implant rupture has been reported to be as high as 55% with 22% of ruptured implants showing the extracapsular spread of silicone [2].

In an attempt to compare Magnet resonance imaging (MRI), with clinical findings of a silicone implant rupture in fifty-five women with 109 implants, it has been reported that less than 30% of ruptured implants were detected clinically, and only 50% of implants diagnosed clinically as intact were complete at MRI [4].

In this case report, we present a 53-years-old woman who experienced lymphadenopathy, as one of the complications after twice breast implantation. This case report has been reported in line with the SCARE criteria [5].

## 2 Case presentation

A 53-year-old woman had a cosmetic silicone gel breast implant in both breasts in 1986 and a replacement with saline gel in 2005. The surgeon noticed small implant ruptures during the replacement of the implants. The patient was assured that there were no remains of the silicone. The patient had no complaints from the breast and observed no change in breast volume. No follow-up took place after the removal of the silicone gel implant.

**Fig. 1:**
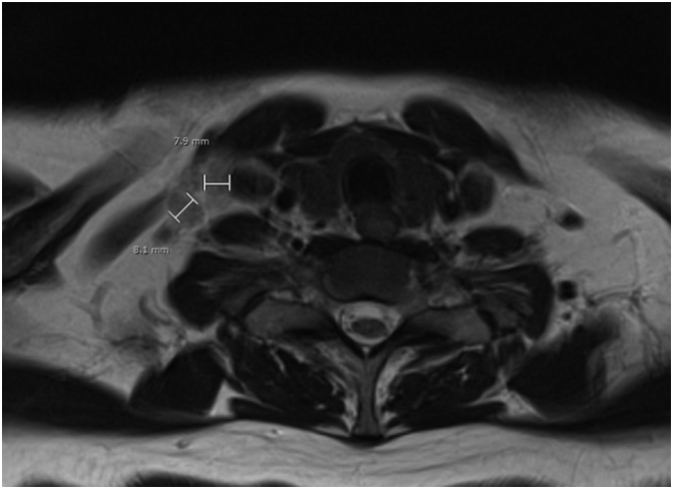
MR scanning shows right-sided cervical lymphadenopathy.

In 2019, the patient had an episode of apoplexia followed by thrombolysis treatment. In CT-angiography one random pathological lymphadenopathy in her right axil was identified and an additional MRI revealed one lymphadenopathy in the right side of the neck (Fig. 1).

**Fig. 2:**
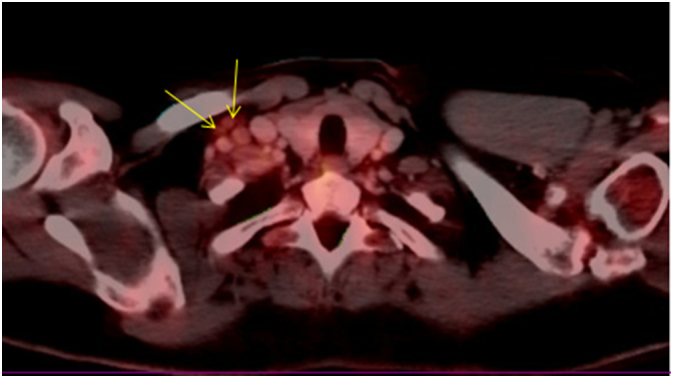
PET scanning shows lymphadenopathy in the right side of the neck.

**Fig. 3:**
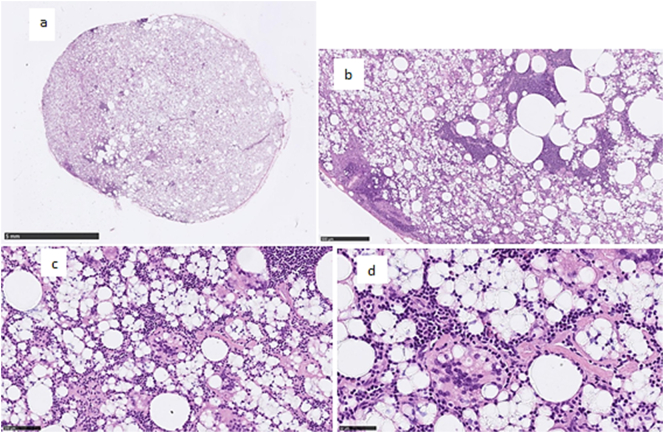
a-d Histological images of numerous histiocytes and giant cells in the lymph node with vacuolated cytoplasm, containing refractile material, consistent with silicone.

A full-body positron emission tomography-computed tomography (PET-CT) scan with radiotracer confirmed a mass on the patient's neck with pathological 18F-fluorodeoxyglucose (FDG) uptake (Fig. 2). A lymph node was removed showing silicone lymphadenopathy and no malignancy (Fig. 3).

## 3 Discussion

Our study represents lymph adenopathy as one of complications of breast implants.

Breast augmentation bears the risk of several complications including bleeding, changes in nipple or breast sensation, as well as rupture, leakage, and migration of silicone [8], [6]. Rupture and silicone migration are implant complications which may vary with implants from different manufacturers based on some unidentified factors such as different shells, gel consistencies, diffusion characteristics, gel chemical compositions, siloxane molecular weighs [1].

There are two main types of implant fillings: Saline (or salt water) and Silicone gel. The saline will be absorbed and naturally expelled by the body if a rupture occurs; meanwhile, the gel in a Silicone implant may remain within the implant shell, or escape to the breast implant pocket [6], [7].

The rupture of silicone breast can manifest with several clinical symptoms and signs such as persistent breast pain, wrinkling of the skin over the implants, asymmetry, scarring, lymphadenopathy, and rarely infection [2]. The rupture can also appear asymptomatic, known as “silent” ruptures [9].

Following implant rupture, silicone can migrate through either hematogenous or lymphatic routes [2]. Silicone lymphadenopathy, which is simply a disposition of silicone in one or more lymph nodes, is a rather late finding reportedly 6–10 years after breast implantation [1].

Leakage and migration of silicone can also cause acute and chronic complications. It can be a local complication resulting from inflammatory foreign body reaction, referred to as “silicone granulomas” or a more distant migration or embolization of silicone including silicone lymphadenopathy [10], silicone pneumonitis, primarily due to subcutaneous injections of silicone [2] or a systemic complication such as granuloma mediated hypercalcemia. All these complications were typically diagnosed 10 to 30 years after silicone placement [10].

The silicone implant rupture can be intracapsular, extracapsular, or “gel bleed”.

Intracapsular rupture is defined as disruption of the implant shell without extrusion of silicone through the fibrous capsule.

Extracapsular rupture is defined as a macroscopic silicone extending beyond the fibrous capsule or some small unpolymerized silicone molecules permeating through the intact elastomer shell of the implant and can travel through the lymphatics, which is called “gel bleed” [2].

Saline implants are considered safer than silicone gel implants [11] and have fewer surveillance needs (because their failure is obvious) [8], but they do contain a silicone shell, which can be rarely associated with pulmonary microemboli. To our knowledge, two cases of chronic silicone pulmonary embolism have been reported as a complication of saline implants involving the lung. In one of these two cases, there was no evidence of rupture or capsular contracture; however, an asymmetry of the breast implants was noted on a careful review of chest imaging [11].

In the reviewed articles, the complications have occurred, while the patients carry their breast implants [8], [2]. However, the study of saline implants has revealed two cases of pulmonary emboli in patients with saline gel implants with silicone shell [11], but no evidence of lymphadenopathy yet.

Our case is a rare and unique presentation of lymphadenopathy and possibly apoplexia as well. There has been no evidence of complications years after the removal of a silicone gel implant. It is not known if the silicone conglomerate found in a cervical lymph node originates from the silicone gel breast removed 15 years ago or from the existing saline implant.

If the silicon conglomerate in the case report is the remains of the removed silicone gel implant, it can confirm the silent rupture and migration of silicon molecules through the lymphatics or blood vessels. As mentioned above, two cases of chronic silicone pulmonary embolism have been presented. However, our case can represent silicone migration from the saline implant with silicone shell.

The fact that the patient has been asymptomatic during all years regardless of silicone gel implant or saline implant, gives no perspective about the approximate time of the silicone migration to lymph nodes or blood vessels. Future innovations may ease the evaluation of a rupture or silicone migration.

## 4 Conclusion

Many studies have shown a correlation between silicone breast augmentation and lymphadenopathy. Rupture and silicone migration are among the complications of silicone and saline breast implant. Having in mind that saline breast implants contain silicone shell, saline breast implants are considered safer than silicone implants. Two cases of pulmonary microemboli have been reported associated with saline implants, no case of lymphadenopathy. The origin of silicone conglomerates in cervical lymph nodes is not known yet.

## Sources of funding

There is no source of funding for my research.

## Ethical approval

Ethical approval not required for case reports in Denmark.

## Consent

The patient gave a verbal consent. The patient wishes to be totally anonymous. A written declaration is provided by the hospital.

## Research registration

Not applicable.

## Guarantor

Christian Grønhøj, Department of head and neck surgery, Rigshospitalet.

## Provenance and peer review

Not commissioned, externally peer-reviewed.

## CRediT authorship contribution statement

Elham Khakbaz, Department of head and neck surgery, Rigshospitalet: Study concept and design, data collection, data interpretation, writing the paper.

Christian Lang, Department of Plastic Surgery, Herlev University Hospital: Editing the paper.

Giedrius Lelkaitis, Department of Pathology, Rigshospitalet: Editing the paper.

Christian Grønhøj, Department of head and neck surgery, Rigshospitalet: data interpretation, design, writing and editing the paper.

## Declaration of competing interest

There is no conflict of interest.
